# Mapping 20 years of irrigated croplands in China using MODIS and statistics and existing irrigation products

**DOI:** 10.1038/s41597-022-01522-z

**Published:** 2022-07-15

**Authors:** Chao Zhang, Jinwei Dong, Quansheng Ge

**Affiliations:** 1 Key Laboratory of Land Surface Pattern and Simulation, Institute of Geographic Sciences and Natural Resources Research, Chinese Academy of Sciences, Beijing, 100101 China; 2 University of Chinese Academy of Sciences, Beijing, 100049 China

**Keywords:** Hydrology, Environmental sciences

## Abstract

As a routine agricultural practice, irrigation is fundamental to protect crops from water scarcity and ensure food security in China. However, consistent and reliable maps about the spatial distribution and extent of irrigated croplands are still unavailable, impeding water resource management and agricultural planning. Here, we produced annual 500-m irrigated cropland maps across China for 2000–2019, using a two-step strategy that integrated statistics, remote sensing, and existing irrigation products into a hybrid irrigation dataset. First, we generated intermediate irrigation maps (MIrAD-GI) by fusing the MODIS-derived greenness index and statistical data. Second, we collected all existing available irrigation maps over China and integrated them with MIrAD-GI into an improved series of annual irrigation maps, using constrained statistics and a synergy mapping method. The resultant maps had moderate overall accuracies (0.732~0.819) based on nationwide reference ground samples and outperformed existing irrigation products by inter-comparison. As the first of this kind in China, the annual maps delineated the spatiotemporal pattern of irrigated croplands and could contribute to sustainable water use and agricultural development.

## Abstract (summary)

**Table:** 

Measurement(s)	irrigation area and distribution
Technology Type(s)	statistics and satellite remote sensing
Factor Type(s)	agricultural irrigation
Sample Characteristic - Organism	agricultural irrigation
Sample Characteristic - Environment	agricultural field
Sample Characteristic - Location	China

## Background & Summary

With the fast-growing population in the world, more grain must be produced to meet the increasing food demand, calling for expanded or intensified agriculture^1,2^. As an important practice of agricultural intensification, irrigated croplands contribute to about 40% of food production using only 18% of croplands globally^3^, but consume 70% of the total available fresh water on the planet^4,5^, exacerbating global and regional water scarcity^6,7^. Thus, explicit information of the spatial distribution and temporal dynamics of irrigated areas is of significance for water management and agricultural planning, as well as the understanding of regional water cycle^8^ and climate change^9,10^.

Irrigation has a long history in China and expanded fast with more and more irrigation infrastructures (i.e., reservoirs and canals) constructed in the last two decades^11^. In China, irrigated croplands account for half of the total cropland area and produce approximately 75% of food and more than 90% of industrial crops^11^. But the sustainable development of irrigation is challenged by episodic weather anomalies, uneven precipitation, and frequent droughts^12^. Long-term intensive irrigation has resulted in water scarcity and environmental problems in some regions, such as groundwater crisis in North China Plain^13^, soil salinization in Northwest China^14^, and local climatic effects (e.g., temperature changes) in Northeast China^15,16^. In the foreseeable future, irrigated agriculture in China is likely to experience pressure in water demand due to unstable weather and extreme climate events, especially in arid and semi-arid areas. Therefore, routinely monitoring irrigated areas can not only facilitate water management and allocation but also evaluate its environmental and climatic effects.

So far, the annual irrigation area is only documented in the statistical yearbook released by the National Bureau of Statistics of China but the explicit information about its location and distribution is absent. The current spatiotemporal extent and dynamics of irrigated croplands in China are still uncertain and existing available maps are usually outdated or with a coarse spatial resolution. During the last two decades, several global irrigation maps have been produced such as the Global Map of Irrigation Areas (GMIA)^17,18^ generated with a 10-km resolution, using approximate irrigation area at national and sub-national scales and multi-source ancillary datasets (i.e., raster maps of land cover and vectorial irrigation maps). Two other global maps based on remote sensing classification methods also emerged subsequently, they were the Global Irrigated Area Map (GIAM)^19^ with a 10-km resolution for the year 2000, and the Global Rainfed, Irrigated, and Paddy Croplands map (GRIPC)^3^ with a 500-m resolution for the year 2005. Further, a couple of time-series irrigation maps at regional or continental scales were also developed by different institutes. For instance, the MIrAD-US^20–22^ (MODIS Irrigated Agriculture Dataset for the US) which utilized a geospatial modelling framework that assimilates irrigation statistics with remote sensing vegetation index to identify the irrigated lands at a 250-m resolution every five years since 2002; the 500-m irrigated dryland map for the US in 2001 produced with remotely-sensed temporal and spectral signatures and a decision tree method^23^; the yearly irrigated area maps in India for 2000–2015 using 250-m MODIS vegetation index, land use/cover data, and a decision tree irrigation model^24^. In addition, several time series 30-m irrigation datasets have been generated using Landsat imagery, environmental variables, and random forest model on the Google Earth Engine platform^25–30^. As for the China region, two national irrigation maps have ever been produced, including the map for the year 2000 generated by downscaling the statistical data to individual pixels based on three irrigation indices^31^, and the map for the year 2016 developed by comparing the water index of cropland to that of adjacent forest^32^. However, these two maps had low accuracies (less than 70%) and involved only single-year information^31,32^, impeding our understanding of the spatial and temporal extent of irrigation area and urgently requesting for such a spatiotemporal dataset.

Here, we presented such a dataset, which resulted from a study attempting to track the spatiotemporal dynamics of irrigated croplands in China from 2000 to 2019^33^. The dataset consists of yearly spatial information describing the extent and distribution of irrigated croplands at a resolution of 500 m, enabling us to find the increase or decrease of irrigated areas across China. Specifically, this study aimed to give a detailed delineation of the producing process of the dataset, including 1) generating provisional irrigation maps (MODIS irrigation dataset based on Greenness Index, MIrAD-GI) by downscaling statistics to potential cropland pixels based on MODIS-derived greenness index; 2) integrating MIrAD-GI with historical irrigation maps into an improved irrigation dataset, using constrained statistics and a synergy mapping method. Further, compared to the previous study which just validated the resultant maps in four typical irrigation districts for three years, more comprehensive validations were implemented based on nationwide ground samples for five years in this study. The data generating process described here was expected to be more explicit, legible, and repeatable with the assistance of accessible Python codes. The final irrigation dataset can be utilized to predict water consumption, assist agricultural planning, and assess regional climatic effects induced by irrigation.

## Methods

China has a typical monsoon climate and is divided into arid, semi-arid, and wet regions. The total precipitation ranges from 3.2–4854.0 mm and the mean annual temperature varies between 4.41–26.33 °C^34^. The unstable climate is prone to result in frequent floods and droughts, which is a threat to agriculture. The total cropland area in China is more than 120 million hectares, accounting for 15% of the global land. Irrigated croplands in this study refer to the areas equipped with irrigation facilities and receive water supply at least once a year, including paddy fields and irrigated drylands. Irrigated croplands mainly spread over flat regions like the North China Plain, Northeast China, the Hetao Plain, the oasis in Northwest China, etc. Irrigation area increased by approximately one quarter in the last two decades^33^ which happened most in Northeast and Northwest China due to the reclamation of croplands.

### Greenness Index (GI) data from MODIS

Vegetation indices (VIs), including normalized difference vegetation index (NDVI), enhanced vegetation index (EVI), and greenness index (GI), are widely used as indicators for irrigation detection^3,29,35–38^ as they represent the amount of green biomass with varied index values responding to the perturbances in vegetation condition^20^. A maximum VI derived from the annual time series can be seen as a proxy for the peak level of photosynthetic activity, the highest biomass, and the possibly densest vegetation canopy^39,40^. The highest annual peak VI for any crops can be attributed to the consistent adequate soil moisture delivered by irrigation during the growing season; thus, it’s common that irrigated crops have higher peak VIs than non-irrigated crops^20,21,23^ (Fig. 4).

Among various vegetation indices, GI is found more sensitive to the irrigation-induced status of crops and can better capture the absolute magnitude of greenness which is an indication of irrigation presence^23^. Gitelson *et al*.^41^ argued that the absorption of light in the green spectrum was high enough to provide GI with a high sensitivity to chlorophyll content but much lower than in the blue and red to avoid saturation. As a result, we selected peak GI as the indicator of irrigation activity. Due to the high temporal resolution and least amount of cloud contamination, the Terra/MODIS surface spectral reflectance data product (MOD09A1) with a spatial resolution of 500 m and an 8-day temporal composite period was used to calculate GI^42^ (Eq. 1).

1 $${\rm{GI}}={{\rm{\rho }}}_{{\rm{nir}}}/{{\rm{\rho }}}_{{\rm{green}}}$$

where ρ_nir_ and ρ_green_ is the near-infrared band and green band of MODIS, respectively. The GI time series were filtered to reduce outliers using the Savitzky-Golay filter^43^.

### Statistical data of irrigation and pre-processing

We collected all available irrigation statistics for 2000–2019 at the provincial, municipal, and county levels released by the National and Provincial Bureau of Statistics (Fig. S1). The statistical data is the only reliable data covering the most irrigated areas in China and has been widely used to assist the mapping of irrigated croplands in existing studies^22,23^. Due to the varied integrities of statistics and adjustments of administrative division in different provinces in the last two decades, we adopted the following measures to produce a consistent statistical dataset. (1) We implemented a linear interpolation to fill the temporal gap using prior and latter years’ data when statistics from one year were absent. (2) We used county-level statistics in priority but adopted municipal or provincial statistics when the finer-level data was unavailable. (3) We unified the statistics of some prefectures and counties whose boundaries or names have ever changed for the whole period. (4) We integrated the multi-source statistics for some provinces like Xinjiang, Heilongjiang, and Hainan, based on geographic locations and administrative boundaries. At last, we got the consistent time series irrigation data and transformed them into shapefile format with ArcGIS.

### Other datasets

Two other datasets were used to facilitate the selection of reference ground samples for validating the resultant maps, they were MOD16A2^44^ and TerraClimate^45^. The 8-day evapotranspiration (ET) data from MOD16A2 with a 500 m resolution was aggregated into monthly mean values to serve as an indicator of irrigation since irrigated croplands usually have a stronger ET than nearby non-irrigated croplands^28,29,46^. In addition, we extracted precipitation data from the TerraClimate which is a dataset of monthly climate and climatic water balance for global terrestrial surfaces^45^, to reflect the climatic background when selecting validation samples.

### Existing irrigation maps and pre-processing

Existing irrigation maps include thematic irrigation products and land use land cover (LULC) products with irrigation information (Table 1). Five thematic irrigation products at global and continental scales were adopted in this study, they were GMIA-m, the extended version of Global Mapping of Irrigation Areas produced by Meier *et al*.^47^ with a spatial resolution of 1 km and a strong correlation with statistical data (r = 0.84); GRIPC, generated using a supervised classification method with remote sensing, climate, and agricultural inventory data at 500 m resolution and had an overall accuracy of 69%^3^; GFSAD (Global Food Security-support Analysis Data) which was a NASA-funded project to provide high-resolution global cropland data and their water use to sustain global food security^48^; IAAA (Irrigated Area Map for Asia and Africa) developed to indicate irrigated and rainfed croplands in Asia and Africa at 250 m resolution for 2000 and 2010^49^; Xiang16 produced for mainland China in 2016 with an overall accuracy of 62%^32^.

**Table 1:** Input spatial datasets for synergizing into the improved irrigation maps.

Data category	Data name	Source	Temporal resolution	Spatial resolution	Spatial coverage	Accuracy	Ref.
Initial irrigation map	MIrAD-GI	This study	2000–2019	500 m	China	—	^20^
Thematic irrigation maps	GMIA-m	https://doi.pangaea.de/10.1594/PANGAEA.884744	2000–2008	1,000 m	Globe	—	^47^
	GRIPC	https://ftp-earth.bu.edu/public/friedl/GRIPCmap/	2005	500 m	Globe	69.0%	^3^
	GFSAD	https://lpdaac.usgs.gov/products/gfsad1kcdv001/	2010	1,000 m	Globe	—	^48^
	IAAA	http://waterdata.iwmi.org/applications/irri_area/	2000, 2010	250 m	Asia and Africa	—	^49^
	Xiang16	Personal communication	2016	500 m	China	62.1%	^32^
LULC maps with irrigation information	NLCD	https://www.resdc.cn	2000, 2005, 2010, 2015, 2018	100 m	China	91.2%	^50^
	CCI-LC	http://www.esa-landcover-cci.org/	2000–2019	300 m	Globe	88.0%	^52^
	GLC_FCS	10.5281/zenodo.3986872	2015, 2020	30 m	Globe	82.5%	^53,67,68^
		10.5281/zenodo.4280923					

MIrAD-GI: MOIDS Irrigated Area Dataset generated by Greenness Index. GMIA-m: the extended version of Global Map of Irrigated Areas from Meier *et al*.^47^, GRIPC: Global Rain-fed, Irrigated and Paddy Croplands, GFSAD: Global Food Security-support Analysis Data, IAAA: Irrigated Area Map for Asia and Africa, Xiang16: Xiang’s irrigation map for China in 2016. NLCD: National Land Cover Dataset of China, CCI-LC: Climate Change Initiative Land Cover, GLC_FCS: Global Land-Cover product with Fine Classification System. LULC: Land Use Land Cover, Ref. indicates references.

Three LULC products were included in this study. The NLCD (National Land Cover Dataset of China) dataset for the years 2000, 2005, 2010, 2015, and 2018 was collected from the Data Center for Resource and Environmental Sciences of the Chinese Academy of Sciences with a 100-m resolution. They were generated through a human-computer interaction method with Landsat imagery and had high overall accuracies of over 90%^50,51^. In the NLCD, paddy fields (class code: 11) which indicated rice and other paddy crops like lotus root were considered as irrigated croplands in this study. Since the NLCD had a five-year interval, we extended the application time to five years including two front years and two subsequent years. E.g., NLCD-2005 was applied for 2003–2007 (Table S1).

The second LULC product was CCI-LC (Climate Change Initiative Land Cover dataset), produced by the European Space Agency (ESA) (yearly since 1992) and consisted of 22 level-1 classes^52^. The explicit definition of irrigated croplands (class code: 20) in CCI-LC indicated irrigated tree crops, shrub crops, and herbaceous crops, as well as post-flooding cultivation of herbaceous crops. The mean accuracy of irrigated croplands in CCI-LC was 88%^52^. The last LULC product was GLC_FCS (Global Land-Cover product with Fine Classification System), developed for the years 2015 and 2020 using a random forest model with Landsat time series and a global training dataset^53^; GLC_FCS had the same classification scheme as CCI-LC and had a mean overall accuracy of 82.5%^53^.

All existing irrigation maps were resampled to 500 m resolution using the nearest sampling method and harmonized to the same coordinate system. We adopted the Albers equal-area projected coordinate system with WGS84 datum as the base projection. In addition, a unified cropland mask derived from the NLCD was used to exclude non-cropland areas in the input datasets for each year (Table S1).

### Generating initial irrigation maps (MIrAD-GI) using a thresholding method

**Fig. 1:**
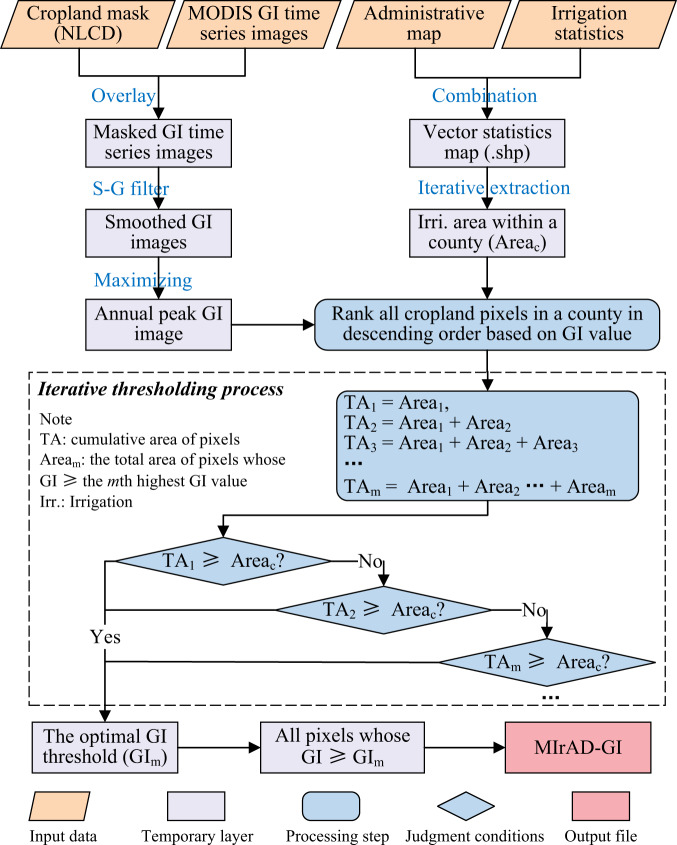
The workflow of generating MIrAD-GI (MODIS Irrigated Area Dataset generated by Greenness Index). “.shp” indicates the shapefile format of the vector irrigation statistics map. The vectorization process is conducted in ArcGIS software. The words in blue color represent image processing. NLCD: National Land Cover Dataset of China, GI: Greenness Index.

We first generated an intermediate irrigation dataset (MIrAD-GI) by fusing remote sensing and statistics. A statistical downscaling or thresholding method^20,21^ was used here to allocate the statistical area to individual cropland pixels. As illustrated in Fig. 1, before thresholding, we overlayed MODIS GI images and the NLCD cropland layer to exclude non-cropland pixels. Then, we calculated the peak annual GI value at the pixel scale based on the smoothed GI time-series images (46 values a year in response to 8-day temporal resolution). Next, within a county extent, all cropland pixels were ranked in a descending order based on GI values. The cropland pixels with the first-highest GI value were counted and their total area was summed up (Area_1_) to match the nominal statistical irrigation area. If Area_1_ was less than the nominal area (Area_c_), then cropland pixels with GI values equal to the 2^nd^-highest GI were counted and their area (Area_2_) was combined and compared with Area_c_. If (Area_1_ + Area_2_) was less than Area_c_, then the iteration process turned to the 3^rd^-highest GI and continued until their total area was closest to Area_c_. The GI value in response to the smallest area gap was the optimal threshold and all cropland pixels with a GI value not lower than this threshold were labeled as irrigated pixels. The above iterative thresholding process was conducted in each county of China to get county-level irrigation maps. Finally, all county-level irrigation maps were mosaiced to constitute the provisional irrigation map (MIrAD-GI). Next, MIrAD-GI was integrated with other irrigation maps into a hybrid dataset for each year (Table S1) using a synergy mapping method, which will be described in the following section.

### Synergy mapping by fusing MIrAD-GI, multiple existing maps, and statistics

Synergistic approaches include two types: agreement-scoring method^54^ and regression method^55^. The former one overlays multiple input products and assigns different scores to each pixel based on the agreement level of the input datasets^54^. As for the latter one, it uses geographically weighted regression and crowdsourced validation data to generate a hybrid map based on existing products^55,56^. We adopted the former one due to the lack of enough validation samples to run a regression model. The key components of the synergy mapping involve (1) quantifying the weight order of input products; (2) overlaying the input products and calculating pixel-wise scores based on the weight order; (3) downscaling the statistical area into individual cropland pixels according to pixel-wise scores.

Determining a suitable weight order of the input products is the first step of the synergistic approach, which relies on explicit accuracy information or empirical judgment from experts. Fritz *et al*.^57^ ranked the input products at a national or regional scale based on the accuracy derived from crowdsourced samples and then integrated them into a hybrid cropland map in 2005. Lu *et al*.^58^ evaluated the accuracies of several existing cropland datasets with a large number of ground truth points when using the synergy mapping method for a hybrid map. When lack of ground samples to assess the accuracy of input products, expert judgment can also play an important role. For instance, when generating a cropland product for sub-Saharan Africa with five land cover datasets, Fritz *et al*.^54^ assigned a higher priority to the regional product derived from higher resolution images or the more recent product. In this study, we used accuracy information and expert judgment fused method to get the weight order of input datasets. We preferred accuracy information as an indicator of priority when it was available but would consider timeliness and spatial resolution and data performance based on empirical judgment. The empirical judgment could play a key role when a regional product derived from higher resolution images or reliable interpretation methods was available but without explicit accuracy information. Timeliness could be considered because the more recent product usually was perceived as more accurate^54^. For instance, the weight order of input products in 2010 was NLCD, CCI-LC, MIrAD-GI, IAAA, and GFSAD. NLCD ranked first due to its highest accuracy (over 90%) and reliability (human-computer interactive interpretation method)^50,51^, followed by CCI-LC whose overall accuracy was 0.88 for irrigated croplands^52^. As an intermediate irrigation product generated by fusing MODIS GI and statistics, MIrAD-GI was closely related to statistical data and covered the approximate irrigated areas^20^ and therefore ranked third. Both IAAA and GFSAD lacked explicit accuracy assessment, so IAAA with a finer spatial resolution (250 m) ranked higher than GFSAD (1 km), based on our empirical judgment rules. Further, through visual inspection, we found that IAAA captured most irrigation hotspots in China and performed better than GFSAD. As a result, IAAA and GFSAD ranked fourth and fifth, respectively.

The second step of the synergistic approach is assigning each pixel a score based on the agreement among input products^59^. In general, one pixel with a higher consensus of irrigation among input products will be more likely identified as irrigation. Various permutations can be obtained based on the agreement of input irrigation datasets and their weight order. For instance, five input datasets adopted in this study were labeled as A, B, C, D, and E according to a descending weight order. In other words, product A ranked first and E last due to their accuracy information or reliability. As illustrated in Table 2, we assigned six agreement levels varied from 0 to 5. Level 5 indicates all five input datasets identify a pixel as irrigated and Level 0 indicates no datasets label the pixel as irrigated. Thirty-two permutations can be obtained based on five input products and a pixel is more likely labeled as irrigated when it has a higher score. The highest score was 31, in response to the highest agreement Level 5, and a score of 0 corresponded to the lowest agreement Level 0. There are multiple scores for other agreement levels due to various arrangements. For example, Level 4 has five combinations with a score ranging from 26 to 30. Based on the weight order, if products A, B, C, and D have the value 1 (irrigation) synchronously, then the score is set 30; when B, C, D, and E have the value 1 simultaneously, the score is 26. Similarly, different scores are assigned according to different combinations (Table 2).

**Table 2:** The agreement ranking score table for five input products.

Agreement level	Score	A	B	C	D	E
5	31	1	1	1	1	1
4	30	1	1	1	1	0
	29	1	1	1	0	1
	28	1	1	0	1	1
	27	1	0	1	1	1
	26	0	1	1	1	1
3	25	1	1	1	0	0
	24	1	1	0	1	0
	23	1	0	1	1	0
	22	0	1	1	1	0
	21	1	1	0	0	1
	20	1	0	1	0	1
	19	0	1	1	0	1
	18	1	0	0	1	1
	17	0	1	0	1	1
	16	0	0	1	1	1
2	15	1	1	0	0	0
	14	1	0	1	0	0
	13	1	0	0	1	0
	12	1	0	0	0	1
	11	0	1	1	0	0
	10	0	1	0	1	0
	9	0	1	0	0	1
	8	0	0	1	1	0
	7	0	0	1	0	1
	6	0	0	0	1	1
1	5	1	0	0	0	0
	4	0	1	0	0	0
	3	0	0	1	0	0
	2	0	0	0	1	0
	1	0	0	0	0	1
0	0	0	0	0	0	0

1 indicates the presence of irrigated cropland and 0 indicates non-irrigated cropland.

**Fig. 2:**
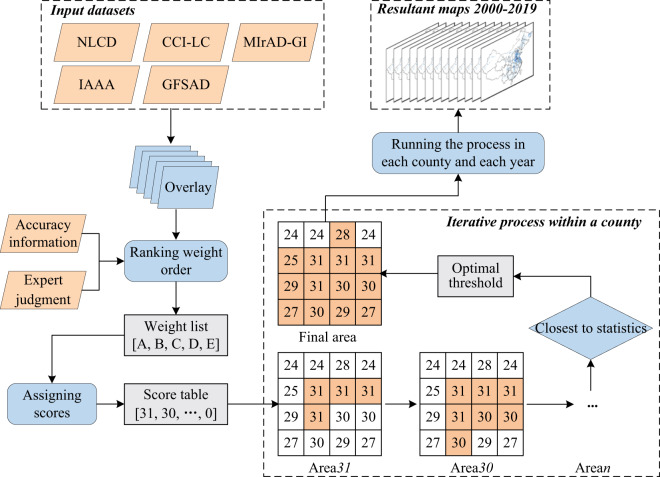
The workflow of generating the resultant maps using a synergy mapping method. NLCD: National Land Cover Dataset of China, CCI-LC: Climate Change Initiative Land Cover, MIrAD-GI: MODIS Irrigated Area Dataset generated by Greenness Index, IAAA: Irrigated Area map for Asia and Africa, GFSAD: Global Food Security-support Analysis Data. Area*n* indicates the area of cropland pixels whose GI value ≥ *n*.

The last step is to downscale the statistical irrigation area to individual pixels based on their score orders. It is an iterative process to allocate the statistical area to the pixels with higher ranking scores automatically until the cumulative irrigation area is closest to the statistical area, like the thresholding process of generating MIrAD-GI (Fig. 1). As Fig. 2 illustrated, within a region (e.g., county and prefecture), the pixels with a score of 31 are counted and their sum area is compared with the statistical area (Area_c_). If the area is less than Area_c_, the pixels with a score of 30 (29, 28, …) are counted and their area is added to match Area_c_ until the cumulative area is closest to Area_c_. After the thresholding process has been done in each county, all resulting county-level maps are integrated into the final map.

## Data Records

The annual irrigation dataset with a 500-m resolution is provided for China during 2000–2019. The dataset is available at the figshare repository in a Geotiff format^60^. The spatial reference system of this dataset is EPSG: 4326 (WGS-1984). All the maps in the dataset are binary maps with 1 indicating irrigated and 0 indicating non-irrigated. The maps can be visualized and analyzed in ArcGIS or QGIS.

## Technical Validation

We adopted two methods to assess the performance of the resultant maps, including pixel-wise validation with nationwide reference data and inter-comparison with existing irrigation products at national and local scales.

### Accuracy assessment using ground truth samples

**Fig. 3:**
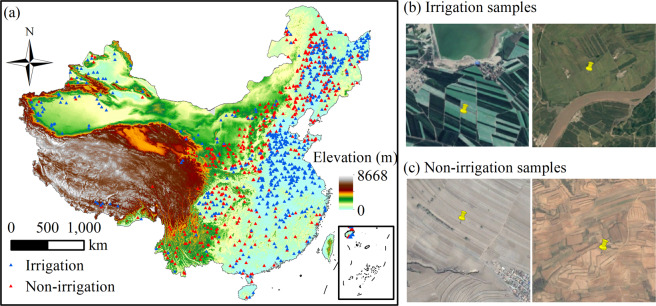
Validation samples and ground-truth images from Google Earth in 2010. (**a**) Nationwide validation samples and the digital elevation model. (**b**) Ground truth images of irrigation samples. (**c**) Ground truth images of non-irrigation samples.

**Fig. 4:**
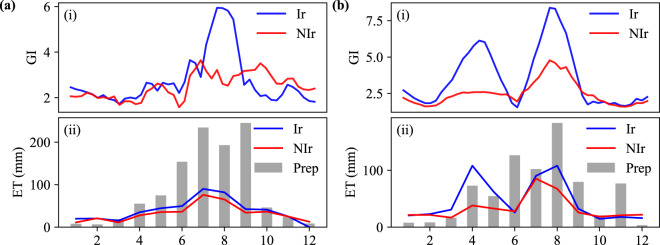
Comparisons of GI (greenness index) and ET of irrigation samples (Ir) vs. non-irrigation samples (NIr) from South China (**a**) and North China Plain (**b**). The monthly precipitation (Prep) is also shown as gray bars (same unit (mm) as ET).

To implement pixel-wise validation, we randomly collected 5,648 ground truth points in five years 2000, 2005, 2010, 2015, and 2019 based on the following three rules. First, croplands close to lakes, rivers, and reservoirs are more likely to be irrigated than those far from water sources. Based on Google Earth images, green homogenous crop fields in the vicinity of water were initially labeled as irrigated (Fig. 3b). Instead, croplands far from water and lacking clear evidence of irrigation infrastructures (i.e., ditches and canals) were identified as non-irrigated (Fig. 3c). Second, we examined the water use of crops within each pixel by plotting monthly time series of ET and precipitation (Fig. 4a). Generally, irrigated crops have higher ET than non-irrigated ones in the growing season from the same region^36^, serving as evidence to distinguish irrigated from non-irrigated croplands. For instance, most winter wheat in North China Plain would be irrigated in April when it turned green because the precipitation is not sufficient. Irrigated crops had a higher monthly ET value than rain-fed crops. Specifically, the ET of March to May exceeds precipitation due to the irrigation practice (Fig. 4b). Third, since irrigated crops are usually greener and have a higher peak greenness index than non-irrigated crops^20^ within the same extent, we further compared the peak greenness index between them. In total, 5,648 validation samples were collected for five years (Table 3, Fig. 3a).

**Table 3:** Accuracy assessment of the resultant irrigation maps using validation samples.

Indicator	2000	2005	2010	2015	2019	Mean
Producer’s accuracy	0.802	0.762	0.684	0.690	0.723	0.732
User’s accuracy	0.844	0.827	0.796	0.811	0.816	0.819
Overall accuracy	0.810	0.776	0.732	0.750	0.759	0.765
Kappa coefficient	0.735	0.683	0.639	0.671	0.673	0.680
F1-score	0.822	0.793	0.736	0.745	0.767	0.773
Irrigation sample #	606	613	658	609	602	618
Non-irrigation sample #	502	475	545	539	499	512
Total Sample #	1108	1088	1203	1148	1101	1130

Note that all these indicators are calculated only for irrigated croplands. “#” indicates the count of samples.

We adopted five evaluation metrics, including the producer’s accuracy (PA, corresponding to omission error), user’s accuracy (UA, corresponding to commission error), overall accuracy (OA), Kappa coefficient, and F1-score as indicators of the performance of the resultant maps. As illustrated in Table 3, the mean overall accuracy was 0.765, with a kappa coefficient of 0.680 and an F1-score of 0.773. All of them were higher than the reported accuracies by Zhu *et al*.^31^ and Xiang *et al*.^32^. The highest and lowest accuracy was found in 2000 (0.810) and 2010 (0.732), respectively. It’s worth noting that the maps had a lower PA than UA, corresponding to a higher omission error than commission error. It may be due to the potential underestimation of the irrigated croplands in statistical data since our synergy mapping method relied heavily on statistics. Several studies have argued that irrigation statistics in developing countries are prone to underestimation due to varied reasons including biased sampling method and political factors^47,61^.

### Inter-comparison with existing irrigation products

**Fig. 5:**
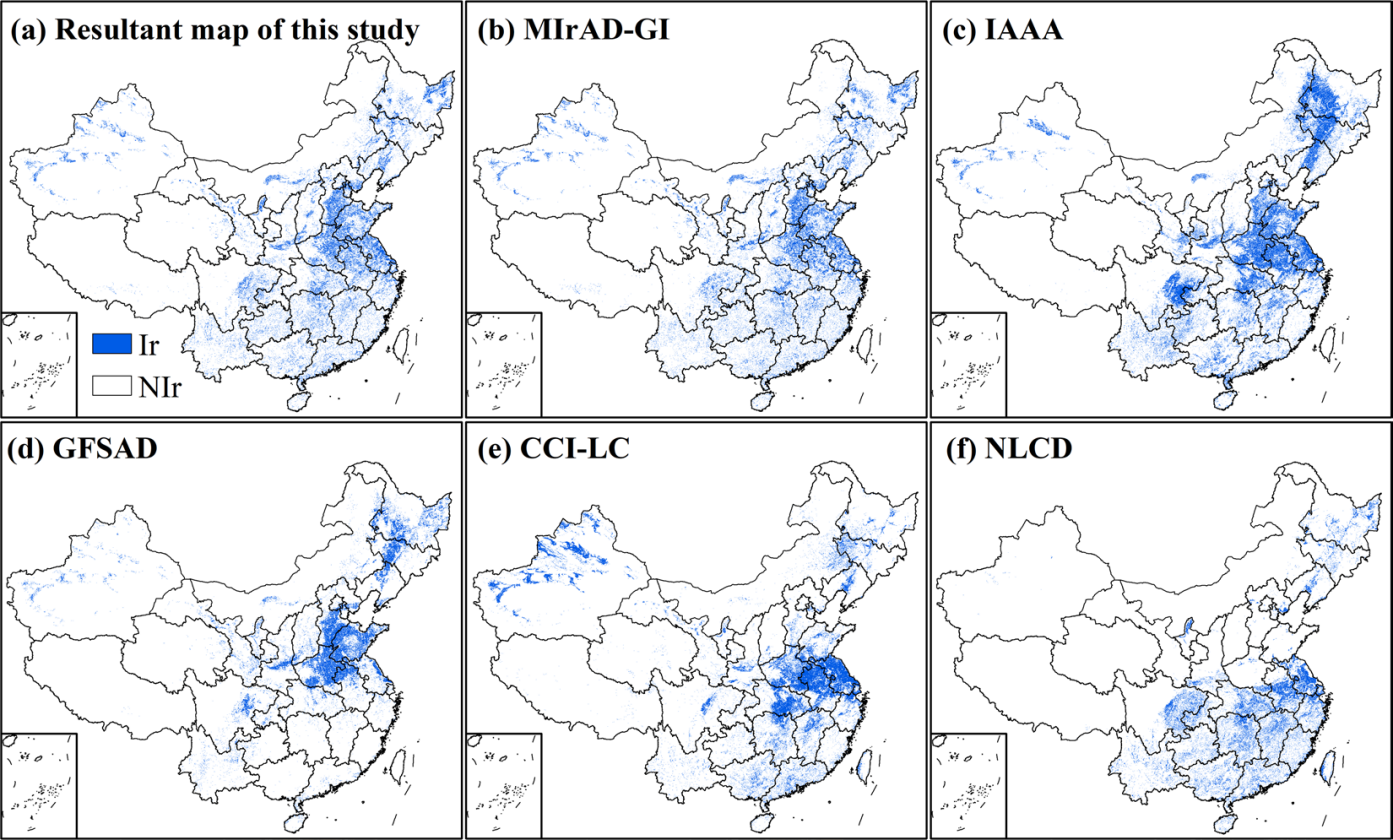
Inter-comparison of the resultant map of this study and the other five existing irrigation maps. The benchmark year is 2010. MIrAD-GI (MODIS Irrigated Area Dataset generated by Greenness Index), IAAA (Irrigated Area map for Asia and Africa), and GFSAD (Global Food Security-support Analysis Data) are thematic irrigation maps. NLCD (National Land Cover Dataset of China) and CCI-LC (Climate Change Initiative Land Cover) are land use land cover products that contain irrigation information. Ir: irrigation, NIr: non-irrigation.

We compared the resultant map with the five input irrigation products for qualitative assessment. Taking the maps in 2010 as an example, we found both similarities and disparities in irrigated areas in these products (Fig. 5). The resultant map matched well with MIrAD-GI and IAAA in most regions and all of them captured the irrigation hotspots, such as Sanjiang Plain and Liaohe Plain in Northeast China, Hetao Plain and Guanzhong Plain in the middle reaches of the Yellow River, North China Plain. Specifically, the resultant map and MIrAD-GI had a highly similar pattern of the distribution of irrigated croplands since both of them were related to GI values and MIrAD-GI played a key role in the synergy process producing an improved dataset. But IAAA suffered from great overestimation, especially in the west of Heilongjiang Province where rainfed corn and soybean were widely planted^62^ (Fig. 5c). GFSAD suffered from great omission in South China (Fig. 5d) and CCI-LC failed to capture the irrigated croplands in Sanjiang Plain and North China Plain (Fig. 5e). NLCD didn’t identify the irrigated croplands in Northern China (Fig. 5f) because its definition of irrigated areas in the NLCD only referred to paddy fields.

**Fig. 6:**
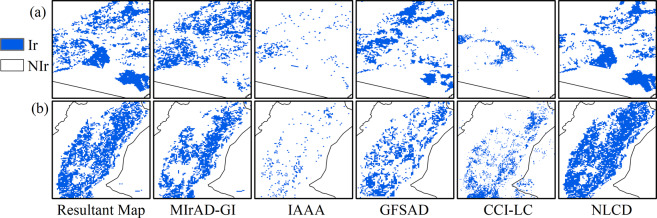
Zoom-in illustration of the resultant map and existing irrigation maps in two irrigation districts. (**a**) The traditional base of paddy fields on the top of Xinagkai Lake (center coordinate: 132.62 °E, 45.51 °N); (**b**) Part of Hetao Plain situated in Ningxia Hui Autonomous Region (center coordinate: 106.48 °E, 38.66 °N). MIrAD-GI: MODIS Irrigated Area Dataset generated by Greenness Index, IAAA: Irrigated Area map for Asia and Africa, GFSAD: Global Food Security-support Analysis Data. CCI-LC: Climate Change Initiative Land Cover, NLCD: National Land Cover Dataset of China.

Specifically, we selected two irrigation districts for further comparison. In an important rice base in Northeast China (Fig. 6a), both IAAA and CCI-LC failed to detect the irrigated areas. The irrigated area in GFSAD was small, which was far from reality. MIrAD-GI was similar to the resultant map but omitted the important paddy fields in the bottom right corner. The south of Hetao Plain (Fig. 6b), a well-known agricultural base along the Yellow River, was not identified by IAAA; the GFSAD covered most but suffered from underestimation, followed by CCI-LC. MIrAD-GI also omitted some irrigated areas and NLCD had a similar performance to the resultant map.

### Uncertainty analysis

Several uncertain factors may have led to some limitations in the resultant maps. First, the statistical data may be inadequate in some regions, especially where the economy is poorly developed. The earlier surveying and sampling method adopted by local statistical bureaus may introduce some uncertainties into the inventory, and the varied political bias of local water managers may also have an impact on the reported area^61^. Furthermore, compared to the irrigated area detected by remote sensing images, statistical data usually suffer from underestimation, because the reported area focuses on irrigated districts equipped with infrastructures but neglects subsistence-level farmland managed by small stakeholders^47^. Nevertheless, statistical data seems to be the only reliable data source on behalf of the most irrigated areas in China.

Second, the hybrid map in this study was highly dependent on source products and their weight order. The uncertainty and bias in source products may be delivered to the resultant map. These errors can be controlled to a large extent through the overlaying and scoring method based on the weight order, but some residuals still existed. Third, our weight sorting method was implemented on the national level, but the accuracy in source products may vary at local scales, which would result in uncertainties. Thus, local adaptive weight ranking orders could be considered in the future if more knowledge about the spatial variations in their accuracies is available. Fourth, although we validated the resultant maps using more than five thousand reference samples extracted through high-resolution Google Earth images with well-defined rules, some bias may still exist due to other factors like georeferenced error and artificial interpretation error. Reference samples from field surveys or irrigation maps from planning authorities may better sustain the validation process and increase the reliability of the final product. Last, there may be some other uncertainties such as the error introduced by irrigated fields where crops do not reach peak GI because of pests, diseases, soil issues (salinity), and other reasons, leading to omission and commission errors.

## Usage Notes

**Fig. 7:**
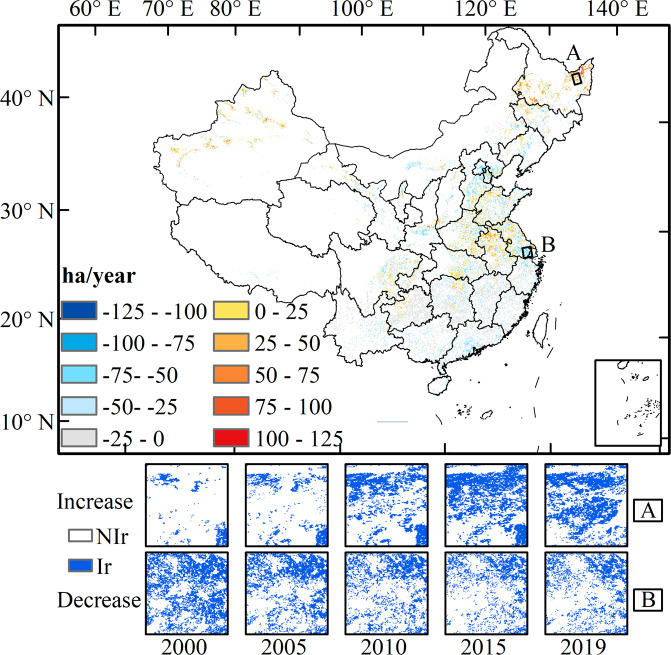
The trends in irrigated cropland area during 2000–2019 at the pixel scale with a resolution of 6 km. The rate of change is derived from linear regression and the 20-year maps. Grids without significant trends (p > 0.05) or having a maximum irrigated area <5% are not shown. (A) and (B) illustrate the zoom-in views of irrigation expansion in Sanjiang Plain and the shrinkage in the Yangtze River delta, respectively.

As an important agricultural practice, irrigation contributes a lot to grain production and food security. Our irrigation dataset with a 500-m resolution for the last two decades is the basic data for various scientific investigations and engineering applications. Our dataset can be used to track the spatiotemporal extent of irrigated croplands and inter-annual change at regional scales (Fig. 7) in China^33^. The water department can use this spatiotemporal dataset to evaluate the performance of irrigation infrastructures since many facilities have been outdated and need to be updated. Policymakers can make better decisions on financial support for water construction according to our dataset. The time-series maps will enable water managers to investigate water consumption^63^ and predict future water requirements, and to better allocate water between different sectors like agriculture and industry. Managers from agricultural sectors can use our dataset to assess planting structures and rotations, predict grain production, and explore the potential yield gap needing to be filled. Under the warming background, our dataset may also be used to evaluate the climatic effects of irrigation, including temperature^15,16^, evapotranspiration^64,65^ wind, and precipitation^66^. Further, the dataset is of significance to help us understand regional hydrological cycles and climate changes.

## Supplementary information

Supplementary Information

## Data Availability

Python code used to generate the irrigation maps is available from the figshare repository^60^. The software used in this work include: ∙ ArcGIS 10.2 ∙ Python 2.7, numpy 1.16.6, pandas 0.19.0, scipy 1.2.3, scikit-learn 0.20.3, matplotlib 1.1.1
