# Supplementary material for: Mapping 20 years of irrigated croplands in China using MODIS and statistics and existing irrigation products
Source: Sci Data. 2022 Jul 15;9:407. doi: 10.1038/s41597-022-01522-z (PMC9287319; doi:10.1038/s41597-022-01522-z)
Supplement: Supplementary file 1 — Supplementary Information [file 41597_2022_1522_MOESM1_ESM.pdf]

Mapping 20 years of irrigated croplands in China using MODIS and statistics and existing irrigation products

Chao Zhang[1,2], Jinwei Dong[1], Quansheng Ge[1*]

1 Key Laboratory of Land Surface Pattern and Simulation, Institute of Geographic Sciences and Natural Resources Research, Chinese Academy of Sciences, Beijing, 100101, China

2 University of Chinese Academy of Sciences, Beijing 100049, China

Corresponding author: Quansheng Ge (geqs@igsnrr.ac.cn)

Table of contents

Figure S1. 2

Table S1... 3

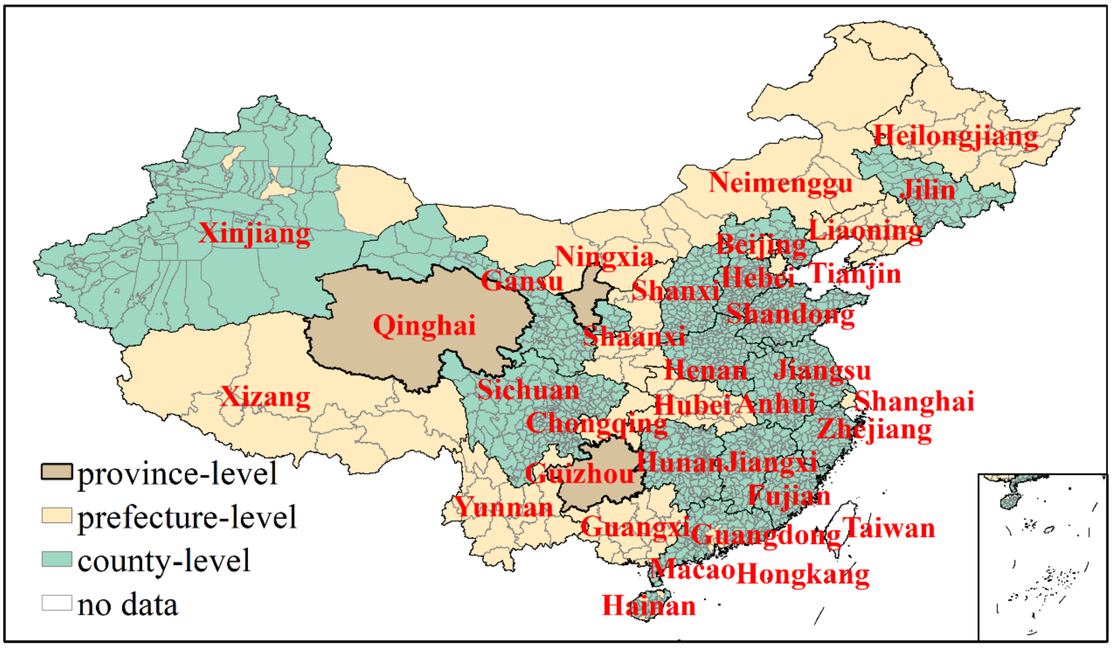

Fig. S1. Statistical data from multiple levels for mainland China.

Table S1. Input datasets and cropland masks used for each year. The temporal resolution of CCI-LC and MIrAD-GI are yearly, and all other datasets have specific benchmark years. NLCD: National Land Cover Dataset of China, CCI-LC: Climate Change Initiative Land Cover, GLC_FCS: Global Land-Cover product with Fine Classification System, GRIPC: Global Rain-fed, Irrigated and Paddy Croplands, IAAA: Irrigated Area Map for Asia and Africa, GMIA-m: the updated version of Global Map of Irrigated Areas from Meier et al., (2018), GFSAD: Global Food Security-support Analysis Data, Xiang2016: Xiang's irrigation map for China in 2016, MIrAD-GI: MOIDS Irrigated Area Dataset generated by Greenness Index. Mask indicates the cropland mask. CP denotes Cropland.

Year	A	B	C	D	E	Mask
2000	NLCD2000	CCI-LC	MIrAD-GI	IAAA2000	GMIA-m	CP2000
2001	NLCD2000	CCI-LC	MIrAD-GI	IAAA2000	GMIA-m	CP2000
2002	NLCD2000	CCI-LC	MIrAD-GI	IAAA2000	GMIA-m	CP2000
2003	NLCD2005	CCI-LC	MIrAD-GI	GRIPC	GMIA-m	CP2005
2004	NLCD2005	CCI-LC	MIrAD-GI	GRIPC	GMIA-m	CP2005
2005	NLCD2005	CCI-LC	MIrAD-GI	GRIPC	GMIA-m	CP2005
2006	NLCD2005	CCI-LC	MIrAD-GI	GRIPC	GMIA-m	CP2005
2007	NLCD2005	CCI-LC	MIrAD-GI	GRIPC	GMIA-m	CP2005
2008	NLCD2010	CCI-LC	MIrAD-GI	IAAA	GMIA-m	CP2010
2009	NLCD2010	CCI-LC	MIrAD-GI	IAAA2010	GFSAD	CP2010
2010	NLCD2010	CCI-LC	MIrAD-GI	IAAA2010	GFSAD	CP2010
2011	NLCD2010	CCI-LC	MIrAD-GI	IAAA2010	GFSAD	CP2010
2012	NLCD2010	CCI-LC	MIrAD-GI	IAAA2010	GFSAD	CP2010
2013	NLCD2015	CCI-LC	MIrAD-GI	IAAA2010	GFSAD	CP2015
2014	NLCD2015	CCI-LC	MIrAD-GI	GLC_FCS2015	Xiang2016	CP2015
2015	NLCD2015	CCI-LC	MIrAD-GI	GLC_FCS2015	Xiang2016	CP2015
2016	NLCD2015	CCI-LC	MIrAD-GI	GLC_FCS2015	Xiang2016	CP2015
2017	NLCD2018	CCI-LC	MIrAD-GI	GLC_FCS2015	Xiang2016	CP2018
2018	NLCD2018	CCI-LC	MIrAD-GI	GLC_FCS2020	Xiang2016	CP2018
2019	NLCD2018	CCI-LC	MIrAD-GI	GLC_FCS2020	Xiang2016	CP2018
